# Supplementary material for: Apicomplexan F‐actin is required for efficient nuclear entry during host cell invasion
Source: EMBO Rep. 2019 Oct 4;20(12):e48896. doi: 10.15252/embr.201948896 (PMC6893294; doi:10.15252/embr.201948896)
Supplement: Supplementary file 1 — Appendix [file EMBR-20-e48896-s001.pdf]

Appendix

Appendix Figure S1. Flow analysis on extracellular parasites and representative images from drug treatment. 2

Appendix Figure S2. Speed profiles of penetrating parasites. 3

Appendix Figure S3. Super resolution microscopy during invasion between wt and chromobody EmeraldFP expressing parasites. 4

Appendix Figure S4. F-actin dynamics during invasion and intensity profiles. 5

Appendix Figure S5. F-actin dynamics and Microtubules during invasion. 6

Appendix Figure S6. F-actin and Microtubules colocalisation during invasion. 7

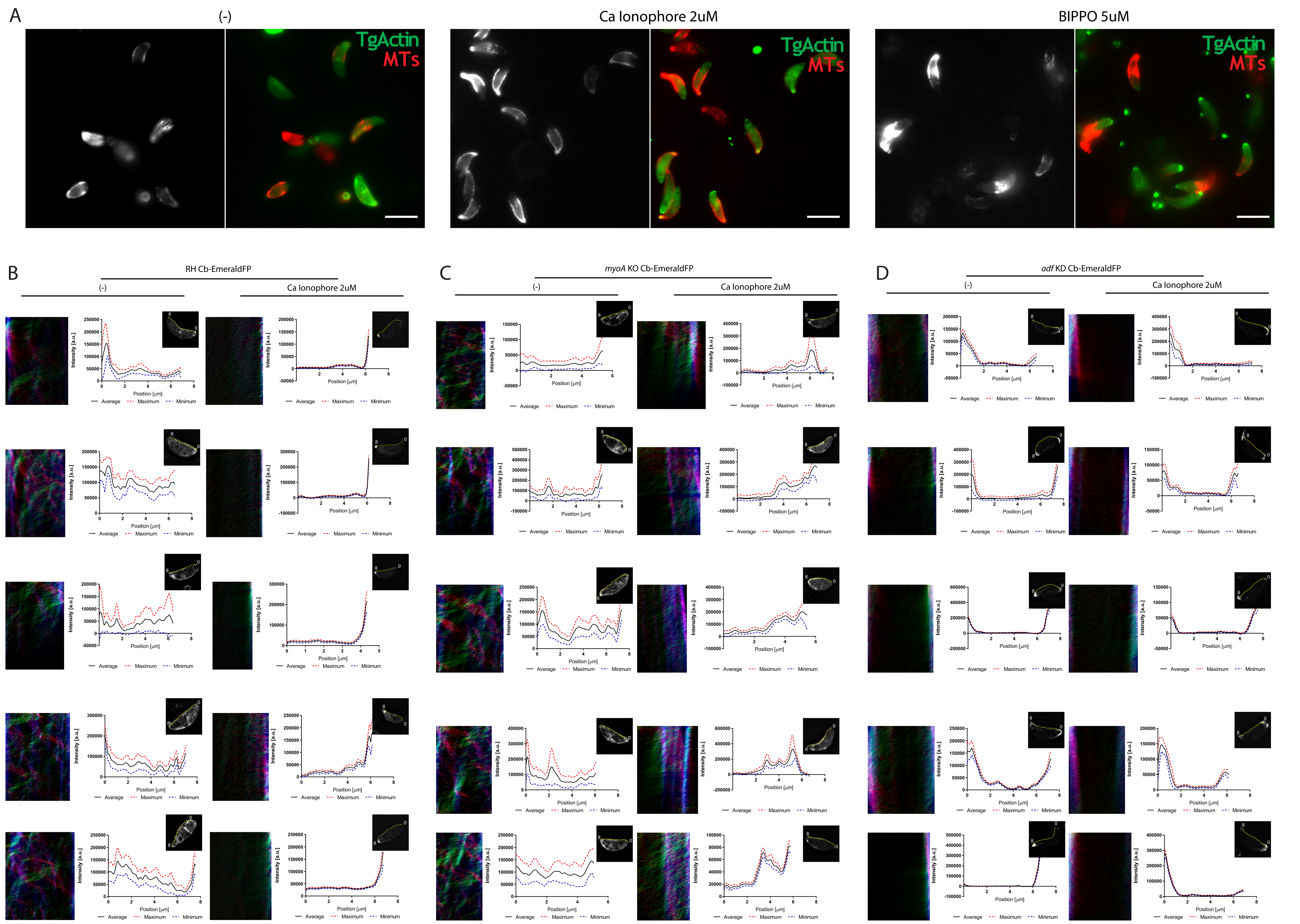

Appendix Figure S1. Flow analysis on extracellular parasites and representative images from drug treatment.

A. Representative images from wild type parasites with calcium ionophore and BIPPO treatment. Scale bar represents 5 µm.

B. Kymograph flow analysis examples done in RH Cb EmeraldFP parasites with and without Ca2+ Ionophore treatment. Before treatment, the parasite appears to show dynamic actin behaviour across the entire periphery, yet upon addition of Ca2+ Ionophore strong actin accumulation can be observed at the apical tip as represented in the kymograph measurement. The colour-coded kymograph represents forward movement (red), backwards movement (green) and static (blue).

C. Kymograph flow analysis examples done in myoA KO Cb EmeraldFP parasites with and without Ca2+ Ionophore treatment. Before treatment, the parasite appears to show dynamic actin behaviour across the entire periphery similar to wt. Upon addition of Ca2+ Ionophore actin can be observed at the periphery, covering approximately half of the parasite peripherical length as represented in the kymograph measurement. The colour-coded kymograph represents forward movement (red), backwards movement (green) and static (blue).

D. Kymograph flow analysis examples done in adf KD Cb EmeraldFP parasites with and without Ca2+ Ionophore treatment. Before treatment, the parasite appears to lack dynamic actin behaviour across the entire periphery unlike wt and myoA KO, upon addition of Ca2+ Ionophore no further change is detected. The colour-coded kymograph represents forward movement (red), backwards movement (green) and static (blue).

A

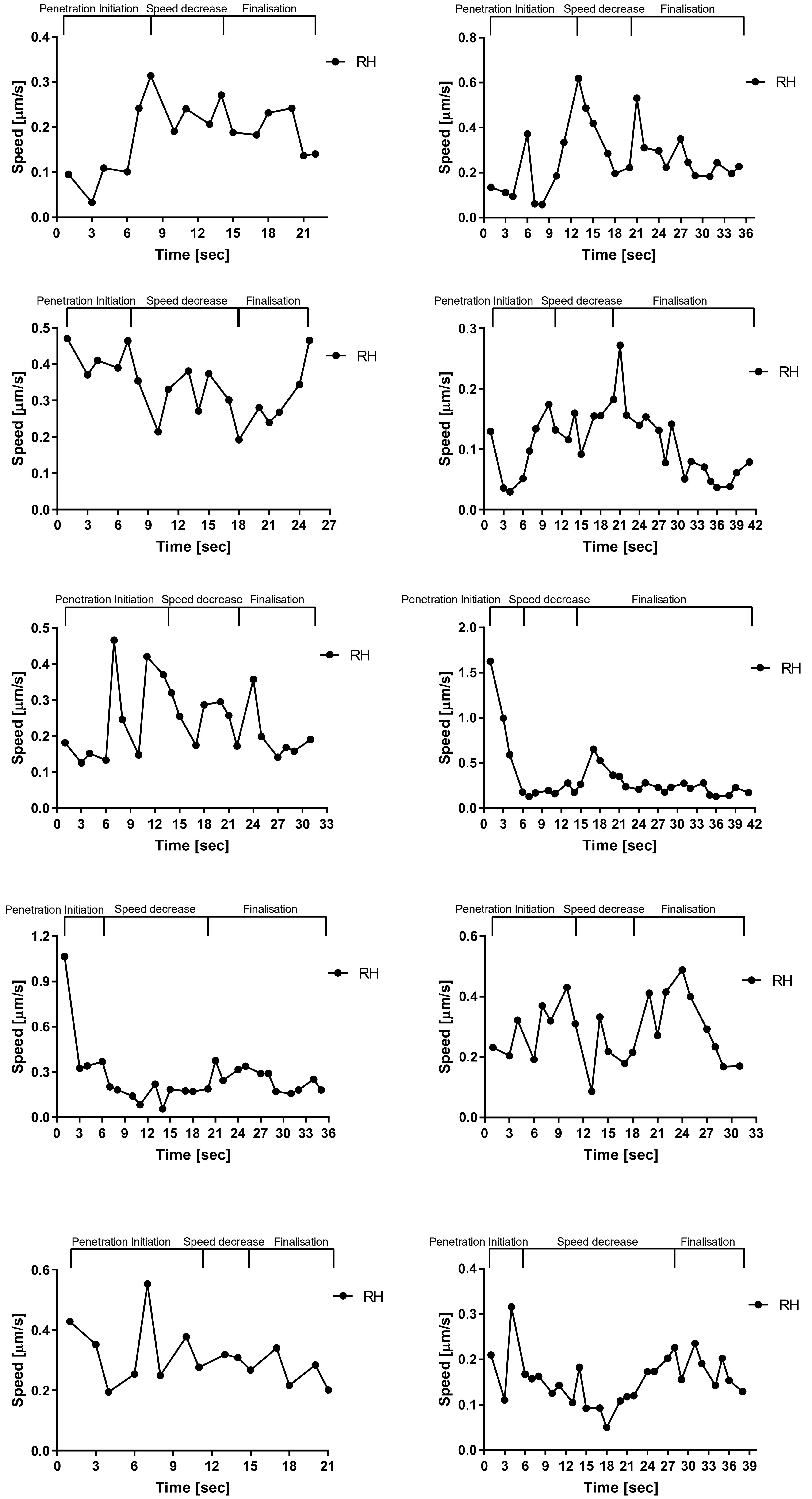

Appendix Figure S2. Speed profiles of penetrating parasites.
10 examples of speed profiles of penetrating parasites as shown in Figure 2.

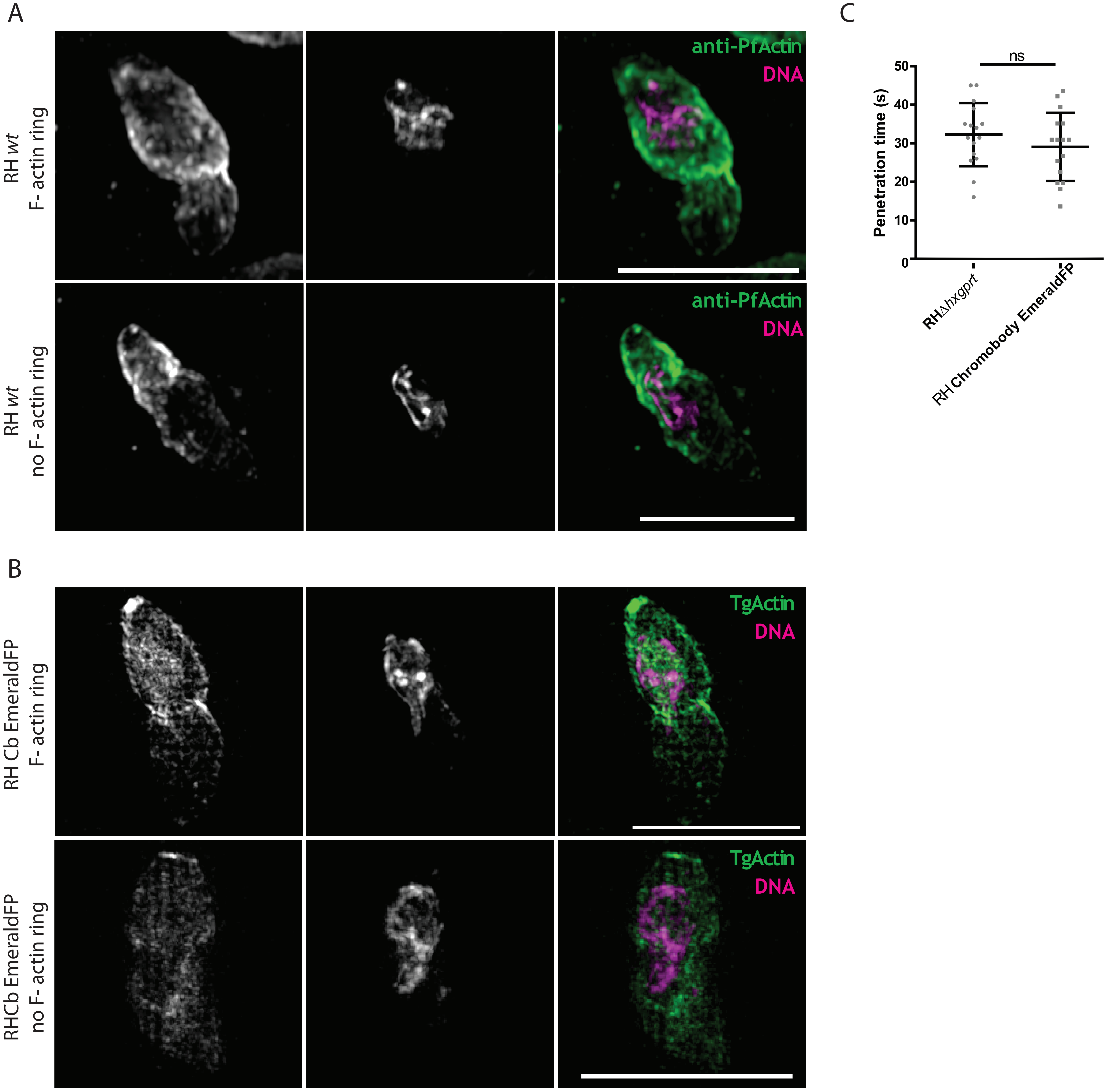

Appendix Figure S3. Super resolution microscopy during invasion between wt and chromobody EmeraldFP expressing parasites.

A. SR images depicting wild type parasites using anti-Pfactin antibodies. In the upper panel a case with an actin ring is shown. In the lower panel, no F-actin ring can be observed.

B. SR images depicting wild type parasites using chromobodies against actin. In the upper panel a case with an actin ring is shown. In the lower panel, no F-actin ring can be observed.

C. Comparison of invasion speeds of RH wild type parasites and parasites expressing Cb-EmeraldFP. 15 movies of each strain were analysed in FIJI. One-way ANOVA analysis was performed for each graph.

Data information: Error bars represent standard deviation. Scale bar represents 5 µm.

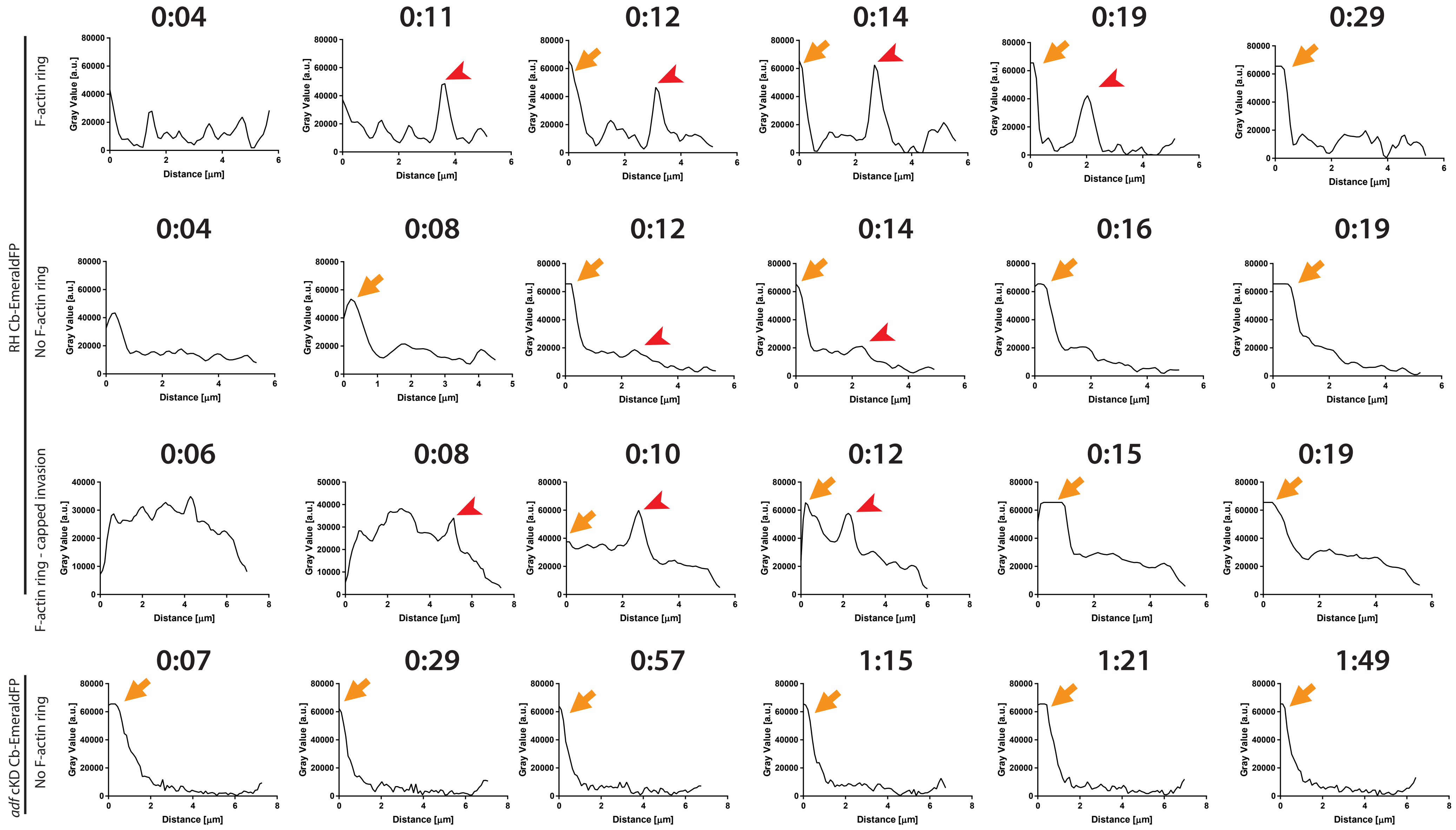

Appendix Figure S4. F-actin dynamics during invasion and intensity profiles.
Intensity plot profiles on Time-lapse analysis of invading RH Cb-EmeraldFP parasites into HFFs. Analysis performed as in Figure 3. The plot profile for wildtype parasites forming F-actin ring at the junction shows two distinct peaks. Accumulation of F-actin at the posterior pole corresponds to the orange arrowhead, while F-actin at the TJ corresponds to red arrowhead. In case of invasion events without formation of an F-actin ring only posterior accumulation can be detected. An F-actin ring is also still formed on rare events such as capped invasion. During invasion of adf cKD parasites (bottom panel) no F-actin ring is formed. Similar to RH parasites F-actin accumulates at the posterior pole (orange arrow).

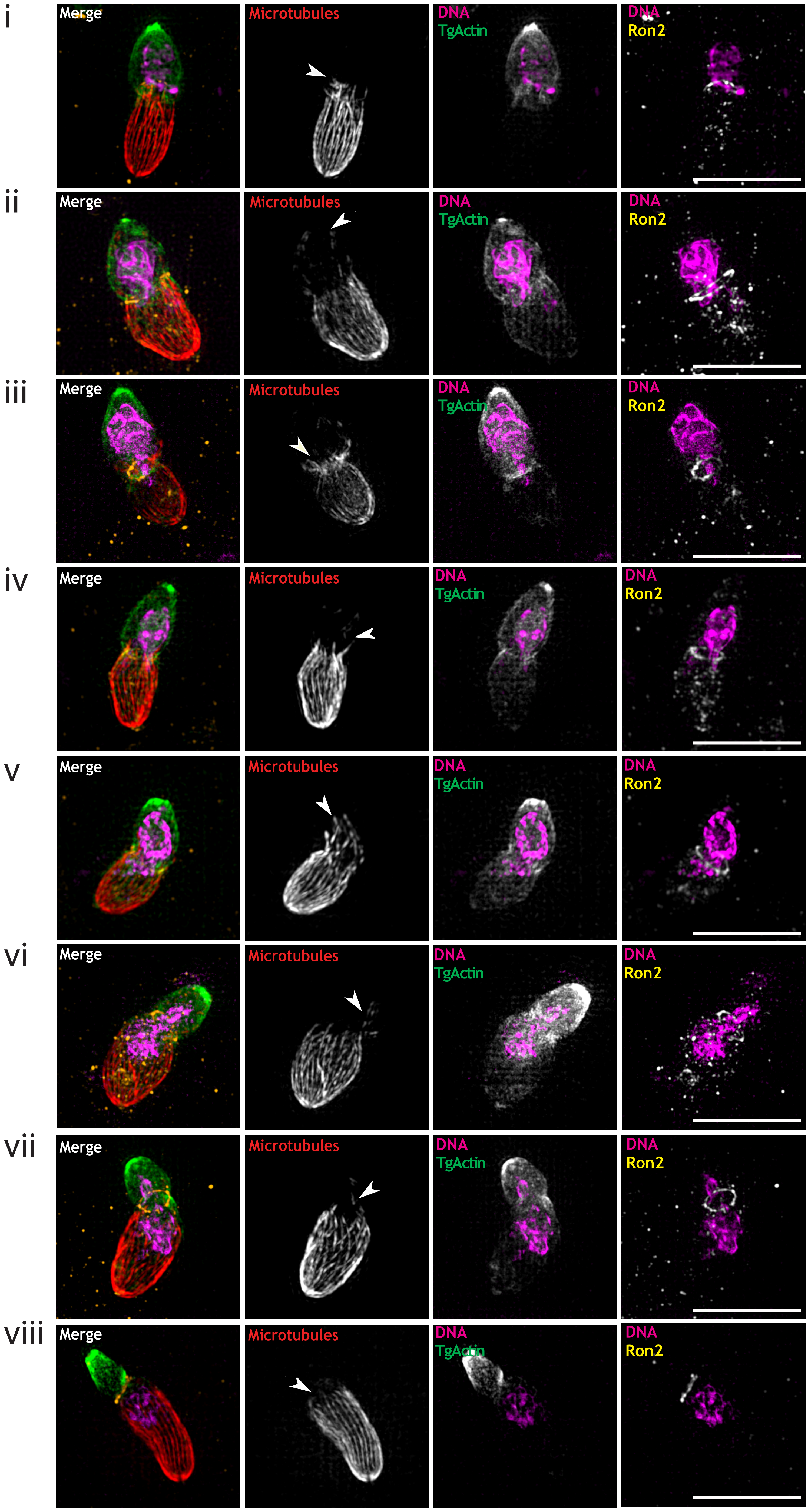

Appendix Figure S5. F-actin dynamics and Microtubules during invasion.
SR images of invading parasites in different stages of invasion. The gallery shows invasion in advancing steps. The MTs are deformed during invasion as show across the gallery (white arrow). In certain cases, such as (ii-iv), the MTs seem to extend further than usual closely aligned to the nucleus. F-actin forms a mesh that is more prevalent in the portion of the parasite that is still outside. The nucleus is constricted when it is going through the TJ (iv-viii). SR-SIM images showing stages of invading wt parasites. SiR-tubulin staining for microtubules (in red) was performed prior to fixation to specifically label microtubules in the parasite. F-actin (in green), Ron2 (in yellow) and DNA (in magenta) were labelled after fixation.
Data information: Scale bar represents 5 µm

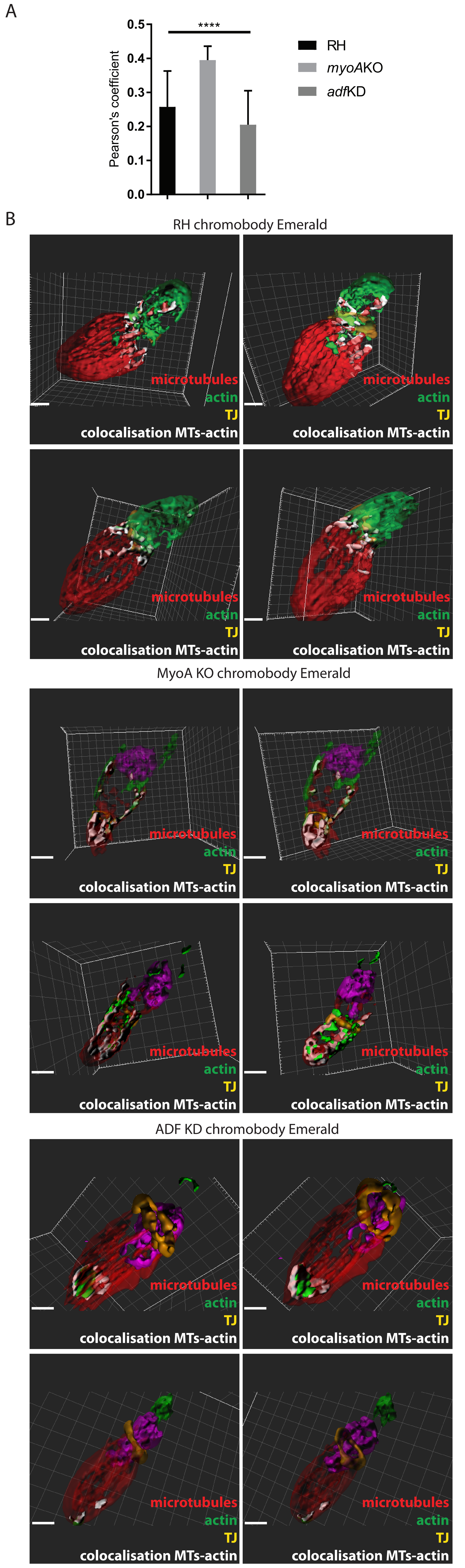

Appendix Figure S6. F-actin and Microtubules colocalisation during invasion.

A. Colocalisation coefficient during invasion of MTs and actin for RH, MyoAKO and ADFKD chromobody emerald . Three biological replicates were analysed with a minimum of 25 parasites across the three replicates. Statistical analysis was done by ordinary one-way ANOVA, using Bartlett's test. ****p-value 0.0002. Error bars represent standard deviation.

B. Representative examples of colocalisation near the TJ area. Green represents F-actin; red for MTs; yellow for TJ; magenta for the nucleus and, white for colocalisation between MTs and actin. Images were rendered via the Imaris software using SR-SIM microscopy images. 36 images were analysed for RH and 20 images each for myoAKO and adfKD using the Imaris software and the Pearson's coefficient was calculated by with the colocalisation module of the same package. Scale bar represents 1 µm.
